# Progressive multifocal leukoencephalopathy in the absence of immunosuppression

**DOI:** 10.1007/s13365-017-0592-2

**Published:** 2017-11-14

**Authors:** Benjamin E. Zucker, Sybil R. L. Stacpoole

**Affiliations:** 1 Imperial College School of Medicine, South Kensington Campus, London, SW7 2AZ UK; 2 Jesus College, University of Cambridge, Cambridge, CB5 8BL UK; 3 Department of Neurology, North West Anglia NHS Foundation Trust, Peterborough City Hospital, Edith Cavell Campus, Bretton Gate, Bretton, Peterborough, PE3 9GZ UK

**Keywords:** Neurovirology, Progressive multifocal leukoencephalopathy, JC virus

## Abstract

A 69-year-old woman presented with a cortical hand syndrome progressing over several weeks. MRI brain showed characteristic appearances of progressive multifocal leukoencephalopathy (PML), confirmed by detection of the JC virus in CSF, despite the absence of any evidence of immunosuppression. Treatment with mirtazapine, mefloquine and cidofovir did not affect the progression of the disease, which was fatal within 7 months of presentation. This report adds to the small case literature that suggests that PML can occur in immunocompetent people, albeit extremely rarely.

## Introduction

Progressive multifocal leukoencephalopathy (PML) is potentially fatal demyelinating encephalopathy of gradual onset caused by the John-Cunningham virus (JCV). JCV is common in the general population with serum antibody to the virus being detectable in up to 80% of adults. Primary infection is usually asymptomatic and leads to indefinite survival of the virus in the kidney (Sabath and Major 2002). In conditions of immunocompromise, reactivation of JCV may occur leading to PML. Human immunodeficiency virus (HIV) infection is the most typical context in which JCV reactivation, and subsequent development of PML, occurs. However, another more recent but relatively common situation in which JCV reactivation and consequent development of PML can occur, is as a result of treatment of multiple sclerosis (MS) with Natalizumab. Both of these examples are associated with a relatively high occurrence of PML, reaching incidence levels of around 1 in 100 in HIV-infected patients in the years before the development of combined antiretroviral therapy and similarly at present, in patients with MS that are high positive for the JC virus (in peripheral blood) and have been on treatment with Natalizumab for 2 years or more (Pavlovic et al. 2015; Plavina et al. 2014). PML is also rarely associated with a plethora of other conditions, including malignancies; autoimmune diseases, such as Sjogren’s syndrome; granulomatous diseases, such as sarcoidosis; and primary immunodeficiencies such as idiopathic CD4 lymphopenia (Zaheer and Berger 2012). These conditions are associated with PML because of immunosuppressive effects—either through the condition itself or through the treatment of them. However, some cases of PML have been reported in the context of only mild immunosuppression such as in hepatic cirrhosis or liver failure (Gheuens et al. 2010) and very rarely in immunocompetent patients.

## Case report

A 69-year-old right-handed retired head teacher presented with fatigue and left hand weakness of insidious onset over 6 weeks. Her past medical history was unremarkable apart from hypertension and mild COPD. She had a 40-pack-year smoking history but stopped smoking 13 years earlier following the diagnosis of COPD. Approximately once a year, she received steroids to treat COPD exacerbations with the last course being 8 months before presentation. She had travelled to Grenada and California with no ill health during these visits.

On examination, she had mild left facial weakness, global weakness of the left hand, increased tone in the left arm and leg, brisk reflexes and a positive Hoffman’s sign on the left, with withdrawal plantar responses. Sensation was normal.

**Fig. 1:**
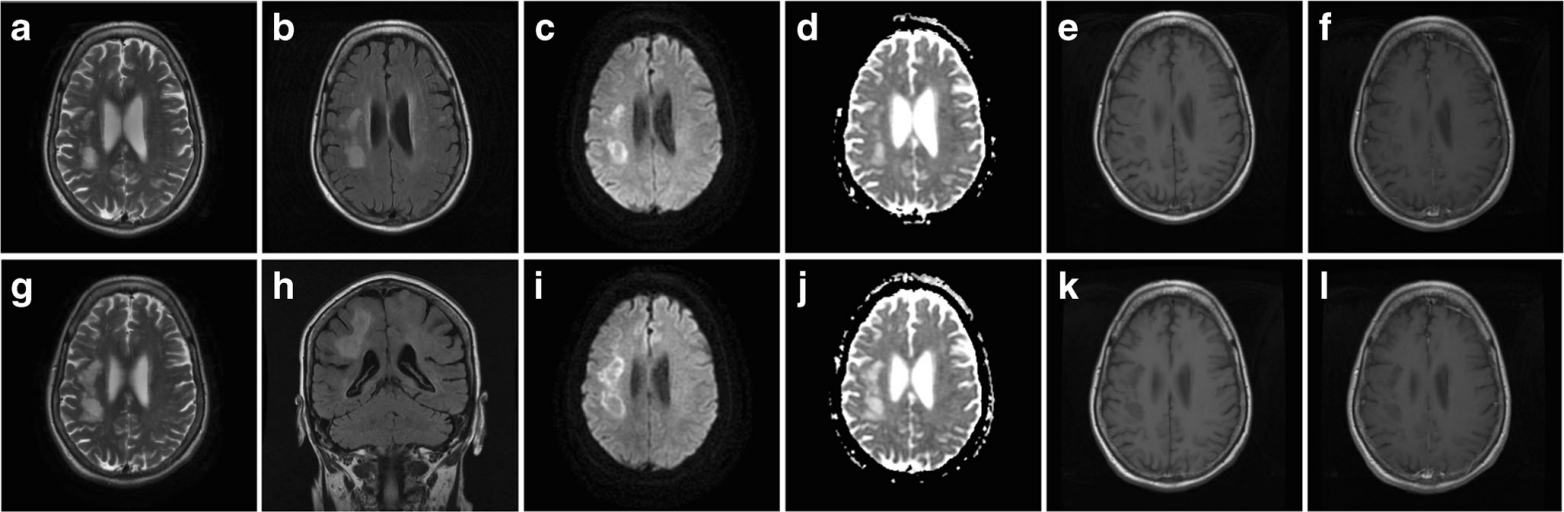
MR imaging of brain demonstrating multifocal areas of high signal on T2 (**a**, **g**) and flair (**b**, **h**) with restricted patchy and peripheral diffusion on the axial DWI sequence (**c**, **i**) matched on the ADC (**d**, **j**). T1 images showed low signal (**e**, **k**) and post-contrast (**f**, **l**) there was no enhancement. The imaging changes progressed over a one-month interval (**a**–**f** followed by **g**–**l**)

MRI of brain revealed multiple T2-hyperintensities in the right corona radiata with restricted diffusion in a patchy, peripheral pattern on diffusion weighted imaging and matched low signal on the apparent diffusion coefficient sequence. Follow-up imaging 4 weeks later showed progression of these changes without contrast enhancement (Fig. 1). There was no hypointensity on susceptibility weighted imaging. An incidental 7 mm diameter aneurysm of the middle cerebral artery was also found. MRI of cervical spine was unremarkable.

The imaging findings and history of gradually progressive weakness were felt to be strongly suggestive of progressive multifocal leukoencephalopathy (PML), despite the fact that she had no risk factors for HIV and had no history of immunosuppression.

CSF PCR detected JVC and confirmed the diagnosis of PML. The rest of the CSF constituents were normal (protein 0.35 g/L, glucose 3.5 mmol/L, white cell count less than 2/μL). HIV-1 and HIV-2 testing was negative. FBC, ESR (2 mm/h), CRP, renal, liver function, B12 and folate, blood film and LDH were all normal. Hepatitis B and C testing was negative. A CT body did not reveal evidence of an occult malignancy.

Serum electrophoresis showed a polyclonal increase in IgA, a finding which is associated with chronic mucosal inflammation such as in COPD. Immunoglobulin measurements were IgG 12.89 (6.00–16.00 g/l), IgA 6.31 (0.80–4.00 g/l) and IgM 0.73 (0.50–2.00 g/l). IgG subclasses 1–4 were also normal.

She was referred to immunology to assess whether she might have an underlying immunodeficiency. Phenotypic characterisation of peripheral B and T lymphocytes by flow cytometry (of blood) was normal (Table 1). Extensive immunological assessment including human T lymphocyte virus (HTLV), functional assessment of her T cells, T cell spectrotype, T cell receptor excisions circles (TRECS) count (which allows assessment of the thymic output of T cells) and in vitro cytokine production did not reveal any underlying immunodeficiency. There was no family history suggestive of immunodeficiency, although one of her two brothers had died at the age of 8 from disseminated TB.

**Table 1:** Lymphocyte flow cytometry

Lymphocyte phenotype	Total (normal range)	Percent
CD3	1.89 (0.70–2.10 × 10^9^/l)	74
CD4	1.56 (0.30–1.40 × 10^9^/l)	61
CD8	0.28 (0.20–0.90 × 10^9^/l)	11
CD19	0.26 (0.10–0.50 × 10^9^/l)	10
CD56	0.41 (0.09–0.60 × 10^9^/l)	16

On the basis of the limited current literature, she was started on treatment with mefloquine 250 mg daily for 3 days initially, followed by 250 mg once a week plus mirtazapine 30 mg once a day. Her symptoms continued to progress such that she lost the use of her left arm after a further month, and the progression of weakness in her left leg led to the use of a wheelchair around 3 months later. After review by the immunologists, she was also commenced on cidofovir 5 mg/kg once weekly for 2 weeks, then continued at fortnightly intervals, with no apparent response after 6 weeks of treatment at which point it was discontinued. She developed a dysarthria 7 months after initial presentation and died shortly after that.

## Discussion

The clinical features of PML result from lytic infection of oligodendrocytes by JCV, typically as a result of failure of the T cell-mediated immune response, whose role it is to prevent JCV reactivation and subsequent viraemia (Gheuens et al. 2010). This, therefore, is the basis of the thinking that progression to PML requires immunosuppression.

Although PML is relatively commonly seen in immunosuppressed patients, it has, very infrequently, been reported in both mildly immunocompromised (Zaheer and Berger 2012) and apparently immunocompetent (Chang et al. 2007) patients. In the 1980s, it was thought that up to 5% of patients with PML had no discernible immunosuppression (Brooks and Walker 1984). However, understanding of the mechanisms of immune dysfunction has since improved greatly, indicating that many of these patients may have been suffering from immunodeficiencies that were, at that stage, undetectable.

There are two different types of JCV: the archetypal form which is transmitted between people in childhood and resides in the kidney and the neurotropic type which evolves from the archetype and causes PML. It has been proposed that the mutations that transform the archetype virus into the neurotropic form occur in the white blood cells of the bone marrow. These cells are then the source of haematogenous spread of JCV to the brain (Wollebo et al. 2015). It has been postulated that a period of dysfunction in the immune surveillance of the virus’ life cycle could be sufficient to allow the necessary viraemia to occur in otherwise immunocompetent patients (Grewal et al. 2016). It is possible that a transient period of immune suppression may result in this dysfunction in immune surveillance (Chang et al. 2007). Considering this, the history of occasional use of oral steroids to treat exacerbations of COPD may be relevant to this case. However, the ubiquitous nature of both oral steroid use and past infection with JCV renders the exposure to oral steroids an unlikely explanation of her development of PML.

The diagnosis of PML requires a combination of clinical suspicion, imaging, CSF PCR for JCV and occasionally brain biopsy. MRI of brain typically shows asymmetric demyelination of white matter represented by lesions that are hypointense on T1-weighted images and hyperintense on T2-weighted imaging and fluid attenuated inversion recovery images. A rim of high signal on diffusion weighted imaging is characteristic, as in this case. The lesions rarely show marked contrast enhancement (Shah et al. 2010); however, faint contrast enhancement may be seen peripherally in approximately 10% of CT and 15% of MRI of brain scans (Berger et al. 1998). Furthermore, contrast enhancement is a common finding in PML-IRIS (immune reconstitution inflammatory syndrome) in association with natalizumab treatment. CSF PCR for JCV is useful in confirming the diagnosis as it has a sensitivity of 67–78% and a specificity of 93–100% (Brew et al. 2010). It is important to note that the sensitivity of CSF PCR is not sufficient to exclude PML, so if clinical suspicion is high, further investigation with a brain biopsy may be indicated.

In vitro evidence suggesting that 5-HT2a receptors play a role in JCV entry into glial cells has led to the investigation of serotonin receptor antagonists such as mirtazapine, as treatment for PML (Elphick et al. 2004). Mefloquine has been suggested to act synergistically with mirtazapine, whereby viral infection of glial cells is prevented by mirtazapine and viral replication is inhibited by mefloquine (Christakis et al. 2013). Despite the proposed mechanism of interaction of mirtazapine and mefloquine in treating PML, there is limited evidence for their use. Indeed, mefloquine was found to have no treatment effect on PML in one randomised study (Clifford et al. 2013).

While the role of cidofovir in treating PML is yet unproven there have been reports both that its use may improve survival (Viallard et al. 2007) and that these effects may be increased in immunocompetent patients (Naess et al. 2010). However, the latter point of view is seemingly at odds with the case described above where, unfortunately, cidofovir had no effect on progression, despite the fact that the patient had no demonstrable immunodeficiency. The outcome of this case seem to be congruent with both in vitro data suggesting that cidofovir does not prevent viral replication in neuroglial cells (Hou and Major 1998), and in vivo data showing that cidofovir has no impact on mortality related to the development of PML (De Luca et al. 2008).

This case demonstrates the possibility that PML may very rarely occur in apparently immunocompetent individuals. Furthermore, the disappointing lack of efficacy of the currently available treatments for this condition is highlighted in this case. This is further exemplified by the high 3-month mortality rate of between 20 and 50% (Brew et al. 2010), which is most noted in those without any reversible immunodeficiency.
